# Invasive Hemodynamic Validation of the Novel HFpEF-JH Risk Score

**DOI:** 10.1016/j.jacadv.2026.102613

**Published:** 2026-02-23

**Authors:** Yael Wollstein, Ryan Sachar, Michael Randazzo, Tess E. Allan, John E. Blair, Gene Kim, Jonathan Grinstein, Mark N. Belkin

**Affiliations:** a Section of General Internal Medicine, University of Chicago Medicine, Chicago, Illinois, USA; b Section of Cardiology, University of Chicago Medicine, Chicago, Illinois, USA; c Division of Cardiology, Duke University Medical Center, Durham, North Carolina, USA; d Division of Cardiology, University of Washington, Seattle, Washington, USA

**Keywords:** heart failure, HFpEF, HFpEF-JH score, invasive hemodynamics

## Invasive hemodynamic validation of the novel HFpEF-JH risk score

Invasive hemodynamics obtained through right heart catheterization (RHC), both at rest and with exercise, are the gold standard in establishing a diagnosis of HFpEF. However, current screening and diagnostic practice remains largely reliant on risk scores. The H_2_FPEF and HFA-PEFF scores are validated diagnostic scores combining age, antihypertensive medications, atrial fibrillation history, body mass index (BMI), and E/e’ ratio and systolic pulmonary artery (PA) pressure obtained via echocardiographic measurements to calculate a score,^1^ with the HFA-PEFF score also incorporating natriuretic peptide and additional echocardiographic measurements.^2^ Recently, a new tool for assessing likelihood of HFpEF—the HFpEF-JH score—was introduced. The score incorporates BMI, estimated glomerular filtration rate, left ventricular (LV) mass index, and left atrial/LV ratio.^3^ This score was shown to have better sensitivity and specificity than H_2_FPEF on initial comparison, particularly in obese-phenotype HFpEF.^3^ However, the score has yet to be validated against invasive hemodynamic data.^4^ This study aimed to externally validate the HFpEF-JH score as a screening tool for HFpEF in patients undergoing invasive hemodynamic testing.

Patient data from 2 studies approved by our Institutional Review Board were used for this analysis. The first cohort was comprised of patients evaluated at the University of Chicago HFpEF clinic with signs and symptoms of heart failure and an LV ejection fraction ≥ 50%, excluding those with end stage renal disease or requiring supplementary oxygen due to primary pulmonary disease. The second included patients added to a coronary function testing registry while being evaluated for HFpEF. The registry enrolled patients assessed for coronary microvascular dysfunction for HFpEF workup between 2015 and 2021 and has been previously described.^5^ HFpEF-JH and H_2_FPEF scores were calculated, and patients were classified as “positive” or “negative” using the HFpEF-JH calculator cutoff point (0.828). True HFpEF diagnosis was diagnosed via resting pulmonary capillary wedge pressure ≥15 mm Hg at rest or ≥25 mm Hg with exercise. The groups were compared using the Mann-Whitney U test. Additional analysis was performed isolating patients with BMI ≥30 kg/m^2^, as the HFpEF-JH score performed particularly well in these patients. Receiver operating characteristic curves were generated using nondichotomized score values (H_2_FPEF as an integer score and HFpEF-JH as a continuous score). Receiver operating characteristic /area under the curve (AUC) was calculated using predicted probabilities from a logistic regression including both scores.

A total of 47 patients were included in the study. Baseline characteristics are described in Table 1. All patients underwent resting, supine RHC, with 23 undergoing additional supine, exercise RHC. HFpEF-JH scores were calculated: 31 patients (66%) were included in the HFpEF positive group (median score 0.94) and 16 (34%) in the negative group (median score 0.78). Patients with a positive HFpEF-JH score demonstrated significantly higher resting hemodynamic parameters, including right ventricular systolic pressure (45 vs 31 mm Hg, *P* = 0.002), mean PA pressure (27 vs 19 mm Hg, *P* < 0.001), and pulmonary capillary wedge pressure (18 vs 13 mm Hg, *P* = 0.03). No significant differences in cardiac output, systemic or peripheral vascular resistances, or exercise hemodynamics were noted between cohorts.

**Table 1:** Top: Baseline Characteristics Reported as Median [IQR] Stratified by HFpEF-JH Screening Status (Positive vs Negative). Bottom: Metrics for HFpEF-JH, and Both Scores Combined in the Total Cohort and in BMI ≥30

	Negative (n = 16)	Positive (n = 31)	*P* Value
Age (y)	61.0 [50.0, 63.0]	64.0 [59.0, 71.5]	0.052
Male (%)	4 (25.0)	4 (12.9)	0.525
Race (%)			0.207
Asian	0 (0.0)	1 (3.2)	
Black	11 (68.8)	27 (87.1)	
White	4 (25.0)	3 (9.7)	
>1 race	1 (6.2)	0 (0.0)	
BMI (kg/m^2^)	30.32 [25.30, 33.65]	38.00 [32.30, 42.58]	**0.005**
BSA (m^2^)	1.82 [1.80, 1.93]	2.10 [1.96, 2.48]	**0.011**
Atrial fibrillation (%)	1 (6.2)	9 (29.0)	0.152
Antihypertensives (%)			0.267
0	0 (0.0)	2 (6.5)	
1	7 (43.8)	6 (19.4)	
2	5 (31.2)	12 (38.7)	
3	2 (12.5)	8 (25.8)	
4	1 (6.2)	3 (9.7)	
5	1 (6.2)	0 (0.0)	
LVEF (%)	63.70 [59.77, 67.15]	61.00 [57.02, 64.33]	0.120
LVMI (gr/m^2^)	68.40 [57.38, 90.48]	95.50 [78.00, 105.80]	**0.012**
LA volume (mL)	37.50 [29.10, 47.35]	62.40 [49.35, 86.20]	**<0.001**
LA:LV ratio	0.43 [0.32, 0.50]	0.70 [0.52, 1.09]	**0.001**
Average E/e	9.40 [7.10, 11.10]	11.90 [8.90, 14.00]	**0.019**
PCWP (rest) (mm Hg)	13.00 [8.00, 17.50]	18.00 [14.50, 22.00]	**0.032**
PCWP (exercise) (mm Hg)	30.00 [18.00, 32.00]	30.00 [25.50, 30.50]	0.577
PA mean (rest) (mm Hg)	18.50 [15.75, 22.25]	27.00 [25.00, 34.25]	**<0.001**
Fick CO (rest) (L/min)	5.80 [5.22, 7.42]	7.45 [5.45, 8.74]	0.164
Thermo CO (rest) (L/min)	5.10 [4.31, 6.20]	5.45 [4.72, 6.52]	0.499
PVR (rest) (Wood units)	1.36 [1.10, 1.64]	1.50 [1.13, 2.30]	0.239
SVR (rest) (Wood units)	1,004.0 [882.0, 1,466.0]	1,016.0 [801.5, 1,249.0]	0.510
proBNP (pg/mL)	86.00 [38.50, 168.00]	250.00 [75.00, 688.00]	**0.007**
Mitral regurgitation ≥ moderate, n (%)	0 (0%)	2 (6.5%)	0.540

	H_2_FPEF (Total)	HFpEF-JH (Total)	Combined (Total)	H_2_FPEF (BMI ≥30)	HFpEF-JH (BMI ≥30)	Combined (BMI ≥30)
True positive, n	16	27	13	13	22	11
False positive, n	1	4	0	1	4	0
False negative, n	20	9	23	15	6	17
True negative, n	10	7	11	5	2	6
Sensitivity	0.44	0.75	0.36	0.46	0.79	0.39
Specificity	0.91	0.64	1.0	0.83	0.33	1.0
PPV	0.94	0.87	1.0	0.93	0.85	1.0
NPV	0.35	0.44	0.32	0.25	0.25	0.26
AUC	0.82	0.71	0.85	0.74	0.59	0.77

Statistically significant *P* values are indicated in **bold**.

AUC = area under the curve; BMI = body mass index; BSA = body surface area; CO = cardiac output; LA = left atrial; LV = left ventricular; LVEF = left ventricular ejection fraction; LVMI = left ventricular mass index; NPV = negative predictive value; PA = pulmonary artery; PCWP = pulmonary capillary wedge pressure; PPV = positive predictive value; proBNP = pro–B-type natriuretic peptide; PVR = pulmonary vascular resistance; SVR = systemic vascular resistance.

Using the invasive hemodynamic criteria for HFpEF diagnosis as the reference standard, 27 of 36 patients with hemodynamically confirmed HFpEF screened positive by the HFpEF-JH score, corresponding to sensitivity of 0.75. Among patients without hemodynamic evidence of HFpEF, 7 of 11 screened negative, yielding specificity of 0.64. The positive predictive value was 0.87 and the negative predictive value was 0.44 (Table 1). Using a cutoff of ≥6, the H_2_FPEF score was found to have a sensitivity of 0.44, specificity 0.91, positive predictive value (PPV) 0.94, and negative predictive value (NPV) 0.35. The 2 scores combined were associated with sensitivity 0.36, specificity 1.0, PPV 1.0, and NPV 0.32. In patients with BMI ≥30 kg/m^2^ (n = 34), the HFpEF-JH score (cutoff ≥0.828) yielded higher sensitivity (0.79) but lower specificity (0.33), with PPV of 0.85 and NPV of 0.25. The H_2_FPEF score demonstrated sensitivity of 0.46, specificity of 0.83, PPV 0.93, and NPV 0.25 in those with BMI ≥30 kg/m^2^.

AUC analyses were conducted, with H_2_FPEF AUC 0.818 and HFpEF-JH 0.707. When applied in tandem, the combined models had an AUC 0.845. The same optimal cutoff value for the HFpEF-JH score of 0.83 was identified in our cohort, consistent with the threshold reported in the original study. When applied together, the 2 scores produced sensitivity of 0.39, specificity 1.0, PPV 1.0, and NPV 0.26. AUC analyses showed modest discrimination for each individual score (H_2_FPEF AUC: 0.738; HFpEF-JH AUC: 0.589), with improved performance when used together (AUC: 0.774). A significant difference in resting mean PA pressure was observed between groups (27 vs 21.5 mm Hg; *P* = 0.0485), whereas other resting hemodynamic variables were not significantly different.

In the initial study introducing the HFpEF-JH score, the novel tool was compared with the H_2_FPEF score and shown to better identify individuals with HFpEF, with particular utility in patients with obesity. Using a machine-learning approach, Bermea et al. identified BMI, estimated glomerular filtration rate, LV mass index, and left atrial/LV ratio as key features associated with HFpEF. These structural and metabolic features may be present earlier in the disease course. In contrast, the H_2_FPEF score incorporates clinical manifestations of more established disease, including atrial fibrillation and elevated pulmonary arterial pressures. These differences in score composition may contribute to the observed differences in test results when evaluated against invasive hemodynamic criteria. Nevertheless, although specificity was high with both scores, neither score fully excluded HFpEF, underscoring the continued role of invasive hemodynamic assessment when clinical suspicion remains high.

This study is limited by its sample size, enriched patient population comprising patients suspected to have HFpEF, and the retrospective nature. However, it is meant to assess this novel score in an external, high prevalence cohort to better understand its testing characteristics as compared to the gold standard of HFpEF diagnosis—invasive hemodynamics.

Our findings corroborate the initial HFpEF-JH study in suggesting that the score could improve screening and risk stratification in both obese and nonobese populations. However, given the variable strengths of the H_2_FPEF and HFpEF-JH scores, a combination of these tools may be the most useful next step in improving diagnostic accuracy.

## Funding support and author disclosures

The authors have reported that they have no relationships relevant to the contents of this paper to disclose.
